# Mosquito-Borne Flaviviruses and Current Therapeutic Advances

**DOI:** 10.3390/v14061226

**Published:** 2022-06-05

**Authors:** Xijing Qian, Zhongtian Qi

**Affiliations:** Department of Microbiology, Faculty of Naval Medicine, Naval Medical University, Shanghai 200433, China

**Keywords:** flavivirus, mosquito-borne, antiviral agent, viral nonstructural protein, host factor, therapeutic strategy

## Abstract

Mosquito-borne flavivirus infections affect approximately 400 million people worldwide each year and are global threats to public health. The common diseases caused by such flaviviruses include West Nile, yellow fever, dengue, Zika infection and Japanese encephalitis, which may result in severe symptoms and disorders of multiple organs or even fatal outcomes. Till now, no specific antiviral agents are commercially available for the treatment of the diseases. Numerous strategies have been adopted to develop novel and promising inhibitors against mosquito-borne flaviviruses, including drugs targeting the critical viral components or essential host factors during infection. Research advances in antiflaviviral therapy might optimize and widen the treatment options for flavivirus infection. This review summarizes the current developmental progresses and involved molecular mechanisms of antiviral agents against mosquito-borne flaviviruses.

## 1. Introduction

Mosquito-borne flaviviruses are single-stranded, positive-sense enveloped RNA viruses, which belong to the genus *Flavivirus*, of the Flaviviridae family, and are transmitted by various species of mosquitoes. The typical mosquito-borne flaviviruses with human medical importance include West Nile virus (WNV), yellow fever virus (YFV), dengue virus (DENV), Zika virus (ZIKV) and Japanese encephalitis virus (JEV). Environmental and ecosystem changes have promoted the exposure of mosquito vectors within human populations, thus increasing the infection risks of these flaviviruses [1].

Each year, more than 400 million people are infected by flaviviruses over the world [2]. The manifestations of mosquito-borne flaviviral infections range from mild symptoms of fevers and arthralgia to severe viscerotropic injuries of livers, kidneys or brains [2,3]. Neurotropic viruses such as WNV, JEV and ZIKV are able to cross the blood–brain barrier to infect neurocytes. Viscerotropic viruses such as YFV infect various types of cells and causes injuries, such as hepatitis, in the relevant organs. DENV is capable of infecting endothelial cells and results in potential hemorrhagic manifestations. Although vaccines are available for some of the mosquito-borne flaviviruses, the low coverage in epidemic areas and limited application in specific populations weaken their effects [4]. There is currently no specific antiviral agent commercially available to treat infected patients. The pharmacotherapies for mosquito-borne flaviviruses are carried out only to alleviate the infection symptoms and provide supportive care. Effective, safe and economical antiviral interventions are needed with a high priority. A number of inhibitors against these flaviviruses are under evaluation in in vitro and in vivo studies or undergoing clinical investigations.

The genome of mosquito-borne flaviviruses is about 11 kb in length and encodes a polyprotein, which is subsequently processed into three structural proteins, i.e., capsid (C), premembrane (prM) or membrane and envelope (E), and seven nonstructural proteins, i.e., NS1, NS2A, NS2B, NS3, NS4A, NS4B and NS5 [5]. The virions bind and enter the host cells through interactions with specific receptors, and viral endocytosis and membrane fusion are triggered to release viral genomes into the cytoplasm for the subsequent replication and translation processes, which involve the participation of various viral NS proteins and host factors [6,7]. Most of the current developing antiviral agents against flaviviruses target viral NS3 or NS5 proteins that possess multiple enzymatic activities including the serine protease of NS2B–NS3, the 5′-RNA triphosphatase, the nucleoside triphosphatase (NTPase) and the helicase of NS3, as well as the methyltransferase (Mtase) and the RNA-dependent RNA polymerase (RdRp) of NS5. These key enzymes are indispensable for the productive replication and efficient production of mosquito-borne flaviviruses, thus becoming optimal intervention targets.

This review aims to overview the current research efforts of antiviral agents targeting the key viral or host enzymes and factors against mosquito-borne flaviviruses, clarifying the molecular mechanisms of these inhibitors and discussing their prospects during the therapeutic application.

## 2. Inhibitors Targeting Flaviviral NS3

Flaviviral NS3 is the second-largest nonstructural protein except for NS5 with a molecular weight of 69 kDa. It is a highly conserved protein that is composed of two domains: the protease domain with a trypsin-like serine protease in the N-terminus for viral polyprotein cleaving, and a helicase along with an NTPase in the C-terminus crucial for viral genome replication and RNA synthesis [8,9].

### 2.1. NS2B–NS3 Protease Inhibitors

**Figure 1:**
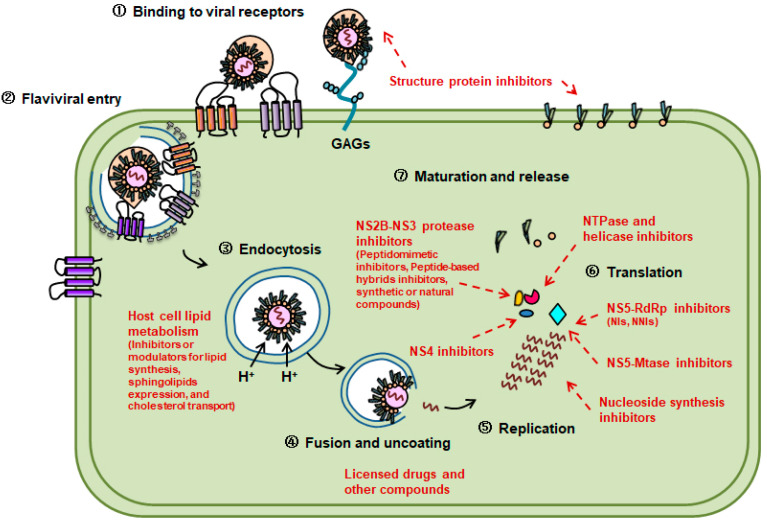
Flavivirus replication cycle and the targeted antiviral strategies. The virion binds to the surface of the host cell through specific receptors and is internalized into the host cell by clathrin-mediated endocytosis. Flavivirus is then uncoated, and its genome is released into the cytoplasm for the subsequent biosynthesis including replication and translation. After that, the virion is assembled and maturated for release. During these processes, various viral proteins and host factors participate in the flavivirus replication cycle, providing multiple antiviral targets for therapeutic interventions. Red words and lines indicate the antiviral agents targeting different viral proteins and host factors during flaviviral infection.

The protease enzymatic functions of NS3 require viral NS2B to be a cofactor, forming an NS2B–NS3 complex to maintain catalytic activity. This complex is critical to the polyprotein processing during flaviviral replication [10]. Therefore, inhibitors targeting NS2B–NS3 could be ideal and attractive therapeutic interventions to combat mosquito-borne flavivirus infections (Figure 1).

Several peptidomimetic inhibitors were identified as NS2B–NS3 protease inhibitors against WNV, DENV and ZIKV [11,12,13]. Agmatine-peptide compounds such as thrombin and furin were inhibitors of trypsin-like serine proteases. Therefore, agmatine-based peptidomimetics were confirmed to be potential suppressors against WNV at micromolar concentrations by a competitive inhibition mode on viral protease activity [14]. Compounds containing retro-peptide, especially the retro-tripeptide series were found to selectively inhibit the activity of NS2B–NS3 against DENV [13]. In addition, the fused bicyclic peptide form of imidazolidinones and pyrrolidines also exerted optimal inhibitory effect on NS2B–NS3 protease against DENV2 through the interaction with Pro132, Ser135 and Tyr150 residues on the complex [15]. Synthesized peptidomimetic compounds consisting of the basic sequence of Abz-Arg-Arg-Arg-Arg-X-X-X-X-Tyr(NO2)-NH2 were identified to have satisfying activity against either DENV1, DENV2, DENV3 or DENV4 NS2B–NS3 proteases by interacting with Asp75 from the N-terminus region [16]. In the in vitro inhibitory assay on the NS2B–NS3 proteases of DENV2 and ZIKV, researchers found that macrocyclic peptidomimetic compounds manifested optimal activities toward these proteases by the noncompetitive inhibition mode at submicromolar concentrations [17,18]. Metallopeptidomimetic compounds that were constituted with a catalytic amino terminal copper and nickel motif were also indicated as effective inactivators of protease activity of WNV and ZIKV NS2B–NS3 [19].

Peptide-based hybrid inhibitors targeting NS2B–NS3 were often designed to bind the catalytic serine by an electrophilic moiety covalently [9]. Peptide hybrids containing thiazolidine or thiophene were also shown to be excellent inhibitors against WNV and DENV2 proteases by interacting with His51, Asp75 and Ser135 residues [20]. Dipeptides containing boronic acid or its derivatives were demonstrated to be potent NS2B–NS3 protease inhibitors toward WNV, DENV2 and ZIKV at nanomolar concentrations with low cytotoxicity in cell cultures [21]. The boronic acid moiety showed substantial increased affinity with multiple NS2B–NS3 proteases of mosquito-borne flaviviruses and formed complexes with them [21]. The X-ray structure analysis confirmed a directly competitive and covalent bound to the viral proteases [21]. Tripeptides composed of the Bz-X-Lys-Phg-NH2 sequence or flexible phenacetyl substituents were found to have good activity against NS2B–NS3 protease of WNV or DENV3 in a cell-based system with low cytotoxicity [22,23]. Synthesized tetrapeptide aldehyde compounds were active protease inhibitors against WNV NS2B–NS3 by interacting with its S1 and S2 pockets, which contained the essential peptide recognition sites [24]. Besides, the peptidyl-aldehyde compounds were also discovered to be strong protease inhibitors against WNV, DENV2 and ZIKV at micromolar concentrations [25,26]. Their aldehyde group inactivated the protease activity by interacting with the Ser135 residue through a covalent mode [26].

A number of synthetic or natural compounds were also screened for their potential activity on flaviviral NS2B–NS3 proteases. Synthesized compounds targeting histone-modifying enzymes were reported to be active suppressors of flavivirus proteases against WNV, DENV and ZIKV and showed significant inhibitory effect on ZIKV infection both in vitro and in vivo [27]. The X-ray assays suggested that this class of compounds worked by binding to an allosteric pocket of NS3 to affect its enzymatic activity [27]. Ivermectin, tyrothricin and selamectin alexidine were also screened out from a compound library and were discovered to suppress the activity of WNV and DENV NS2B–NS3 proteases by either a competitive or a mixed noncompetitive inhibition in the high-throughput screening assay [28]. Aprotinin is a bovine pancreatic trypsin inhibitor with broad-spectrum inhibitions on various serine proteases including NS2B–NS3 proteases of DENV and ZIKV at nanomolar concentrations [29,30].

NS2B–NS3 proteases are attractive targets for antiflaviviral development, since their inhibition led to a direct suppression of viral replication. However, since the active site of the NS2B–NS3 complex is quite shallow, it is a big challenge to develop inhibitors with high potency. Most of the compounds only shared weak binding to the NS2B–NS3 proteases, and the studies basically remained in cellular or animal evaluation. Nevertheless, the advances of modern medicinal chemistry offered large numbers of modified derivatives to be potential ideal candidates for alternatives that might make up future therapeutic regimens.

### 2.2. NTPase and Helicase Inhibitors

The C-terminus domain of flaviviral NS3 protein is responsible for the NTPase and helicase activities. The NTP hydrolysis provides chemical energy, which helps the helicase translocate into the double-helix position for the subsequent activity [31]. Inhibitors targeting NTPase and helicase could hinder the unchaining process, thus interfering in flaviviral replication (Figure 1).

Although the crystal structures of the flaviviral NS3 helicases are short of binding pockets for small-molecular inhibitors, there were still some reported compounds with suppressive activities against the NS3 helicases. Suramin, a synthesized polysulfonated naphthylurea compound, was found to inhibit DENV helicase activity in a noncompetitive manner during enzyme-catalyzed assays [32]. Benzothiazole and pyrrolone were also potent flavivirus inactivators targeting the NS3 helicases of WNV and DENV to impede viral replication in a cell-based system [33]. In addition, the nucleoside compounds of 5′-O-fluorosulfonylbenzoyl esters of inosine (FSBI) and analogs of 1H-benzotriazole and 1H-benzimidazole were also found to be potential inhibitors against WNV, DENV or JEV in the enzyme-based assays [34,35,36]. However, the activity of most inhibitors was quite limited in the cell-based system.

## 3. Inhibitors Targeting Flaviviral NS4

The NS4 of flaviviruses is composed of NS4A and NS4B. Although these two proteins possess no reported enzymatic activity during viral infection, they are critical in viral replication and host cell interaction. Viral NS4A is responsible for the membrane reorganization and autophagy to facilitate the flaviviral replication, and NS4B is involved in the regulatory effect on host cell immune responses against the viral infection [37].

NITD-618 was identified as an effective compound against all four serotypes of DENV by interrupting the formation of viral NS3–NS4B complex at micromolar concentrations [38]. The antiviral activity was specific in DENV compared to other mosquito-transmitted flaviviruses [38]. However, its poor pharmacokinetics in animal models restrained its application. Small-molecular compound of CCG-3394 and CCG-4088 were also found to possess anti-YFV and anti-DENV potency by targeting viral NS4B protein through a replicon-based assay [39,40]. The resistant variants under the treatment of these compounds harbored a mutation in the cytoplasmic loop of viral NS4B, further validating their antiviral target. Lycorine is an alkaloid compound extracted from natural plants. It was demonstrated to have a universal antiflaviviral potency against WNV, YFV and DENV in cell cultures at micromolar levels [41]. The viral titers were alleviated, and viral RNA replication was suppressed under the treatment of lycorine and its derivatives, probably by a direct effect on the 2K peptide between viral NS4A and NS4B.

## 4. Inhibitors Targeting Flaviviral NS5

Flaviviral NS5 is the most conserved and largest nonstructural protein. Since it has multiple functions that are essential and critical for viral replication, it has become an attractive and promising target for the development of antiviral agents. Moreover, recent advances in chemical and structural modifications for compounds with antiviral potency have greatly facilitated the research and development progress for NS5-targeted drugs.

### 4.1. RdRp Inhibitors

The NS5 protein contains an Mtase domain in the N-terminus and an RdRp domain in the C-terminus. The RdRp domain initiates RNA synthesis and is responsible for the productive replication of the viral genome [42]. Flaviviral RdRp is composed of the palm, finger and thumb subdomains, which form a canonical right-hand structure. Since the replication and transcription of human genes do not require RdRp, flaviviral RdRp becomes the most promising targets with favorable safety for the development of antiflaviviral agents (Figure 1) [43].

#### 4.1.1. Nucleoside Inhibitors

Nucleoside analog inhibitors (NIs) are synthetic nucleosides with chemical modifications, which could mimic endogenous nucleosides, thus suppressing their original functions during viral replication. Since the first developed nucleoside analog inhibitors in the late 1960s, more than 25 therapeutic candidates have been approved to treat viral infection. Some of them, especially the ones applied in the treatment of flavivirus infection, such as hepatitis C virus (HCV), are being evaluated for their therapeutic potentials in mosquito-borne flaviviruses [44]. Most NIs target the active site of the polymerase, which is located in the palm subdomain of the RdRp, and terminate viral nucleic acid synthesis by formatting nonfunctional viral RNA chains [45]. The application of nucleoside analogs during mosquito-borne flaviviral infection is likely to reduce the emergence of viral resistant mutants under long-term drug pressure [46].

Several modified NIs were capable of inhibiting the enzymatic activity of flaviviral RdRp in vitro. Two 4′-azidated nucleoside analogs, 4′-azidocytidine (R-1479) and 4′-azido-aracytidine (RO-9187), showed strong anti-WNV activities in vitro [47]. However, their effect seemed to be cell-type specific only in porcine kidney stable cells. GS-441524, a 1′-cyano substituted C-nucleoside exhibited inhibitory effects on YFV and DENV infection under micromolar concentrations in multiple cell lines [48]. 2′-C-Methylated nucleosides were also highly effective against some mosquito-borne flaviviruses [49,50]. INX-08189 was a prodrug of 6-O-methyl-2′-C-methylguanosine, which inhibited DENV2 infection potently, especially when combined with ribavirin in a cell-based system [51]. The tritylated pyrimidine nucleosides such as 2′,5′di-O-trityluridine were demonstrated to suppress YFV and DENV2 replication potently in a cell-cultured system [52]. 2′-C-Methylated nucleoside inhibitors were also reported to have a suppressive effect on ZIKV infection in both immortalized and pluripotent stem-cell-derived neuronal cell lines [53].

In addition, some of the NIs also manifested quite good in vivo efficacy, suggesting their promising application as candidate antiflaviviral drugs. 2′-C-Methylcytidine protected ICR suckling mice from DENV challenges, and hamsters from lethal infection of YFV by suppressing the activity of their relevant viral NS5 polymerase. 7-Deaza-2′-C-methyladenosine possessed optimal antiviral effect on WNV infection, protecting mice from disease progression and lethality even under treatment initiated 3 days after infection [47]. 2′-C-Ethynyl-substituted nucleosides and their derivatives were found to be potent inhibitors of DENV of different serotypes at low micromolar concentrations in multiple cell lines with low cytotoxicity [54,55]. Moreover, one of the compounds known as NITD008 exerted optimal inhibitory effect on WNV, YFV and ZIKV infection both in cell-based systems and mouse models [56,57]. However, its toxicity issues made it fail the preclinical investigation. BCX4430 is an imino-C-nucleoside against a number of mosquito-borne flaviviruses including WNV, YFV, DENV, ZIKV and JEV at micromolar concentrations in in vitro study [58,59]. This compound was also reported to protect hamsters from lethal YFV challenges and mice from ZIKV infection [60,61].

A series of pyrazinecarboxamide derivatives were evaluated as candidate antiflaviviral drugs. T-1106 is a heterocyclic base-modified nucleoside. It was reported that T-1106 manifested quite favorable in vivo efficacy against YFV infection and had a synergistic treatment effect when combined with ribavirin in a hamster model [62]. T-705 (6-fluoro-3-hydroxy-2-pyrazinecarboxamide) also known as favipiravir is a nucleoside analog that exhibited an inhibitory effect on a broad spectrum of flaviviruses including WNV, YFV and ZIKV [63]. Daily treatment with favipiravir provided sufficient protection against fatal ZIKV challenges in IFNAR^−/−^ mice [64]. However, the antiviral activity seemed to be sex dependent with a higher survival rate in female groups probably because of the sex-specific host response.

Some of the NIs have greater clinical significance due to their original clinical applications or research advancements. FDA-approved sofosbuvir is a prodrug of 2′-fluoro-2′-C-methyluridine, which is a potent RdRp inhibitor widely applied in the treatment of chronic HCV infection [65]. This compound was identified to have an inhibitory effect on YFV infection in human hepatoma cells at micromolar concentrations and improved the survival of infected neonatal Swiss mice and adult immunodeficient A129 mice [66]. DENV replication could also be blocked by sofosbuvir [67]. Moreover, it was reported that sofosbuvir inhibited ZIKV replication by targeting RdRp in multiple cell lines and protected immunosuppressed C57BL/6J mice from lethal ZIKV infection [68]. The neuromotor impairment was alleviated by the treatment of this compound, and the viral titers were reduced in the serum and targeted tissues such as brains, spleens and kidneys in ZIKV-infected mice [69]. Furthermore, a prodrug of 4′-azidocytidine called balapiravir, which was an optimal inhibitor in chronically infected HCV patients, was also shown to have potent antiviral activity against DENV of multiple serotypes and strains. This clinically investigated drug was further progressed into clinical research stages to evaluate its treatment efficacy in DENV infection. However, the oral dosage of 1500 and 3000 mg BID of balapiravir achieved no significant effect on DENV patients in viremia kinetics or hematological and biochemical parameters, thus failing the clinical evaluation [70]. Nevertheless, the lessons learned from the failed trials could be helpful for the future development of anti-DENV agents.

#### 4.1.2. Non-Nucleoside Inhibitors

Non-nucleoside analog inhibitors (NNIs) inactivate polymerases by binding to their allosteric pockets or surface cavities instead of the putative active sites to induce a conformational change [71]. Therefore, the allosteric inhibition by NNIs is an attractive strategy for flaviviral RdRp inactivation.

Pyridobenzothiazole-based compounds were found to significantly suppress WNV and DENV RdRp activity and impede viral infection in cell-based systems at low micromolar levels [72]. The involved mechanism was related to the binding of these compounds to the RNA template tunnel during the enzymatic activity, thus locking the polymerase in a closed conformation for further viral RNA synthesis [73]. DENV allosteric N-pocket inhibitors are a class of compounds designed according to the identified “N pocket” around the interface between the thumb and palm subdomains of DENV RdRp. They were found to hinder DENV replication of multiple serotypes in various cell lines [74,75]. However, the binding affinities were not very strong due to the lack of additional contacts with the C-terminal loop of DENV RdRp [76]. Rifapentine, a rifamycin antibiotic, was discovered to suppress both vaccinal and wildtype YFV infection in cell cultures and protected immunodeficient and normal mice from lethal YFV challenges through binding the active site of RdRp domain [77].

### 4.2. Mtase Inhibitors

The N-terminus Mtase is responsible for the methylation of the 5′-RNA caps of genomic RNA to stabilize viral mRNA for efficient translation. The nucleoside analog S-adenosyl-L-homocysteine (SAH) is the methylation product of flaviviral Mtases, thus becoming a natural inhibitor of viral Mtases. Researchers found that SAH together with its derivatives were strong inhibitors of DENV or ZIKV Mtases [78]. Such compounds also included sinefungin, a SAM (S-adenosyl-L-methionine) analog and GMP (guanosine monophosphate). However, these antivirals manifested poor cell permeability for further development.

The thymine-base-containing nucleosides, GRL-002 and GRL-003, were demonstrated to have a favorable activity against WNV Mtase in cell cultures [79]. 5′-Silylated 3′-azidothymidine substituents were observed to be effective elements in the inhibition of WNV and DENV replication through competitive interactions with the active binding site of the flaviviral Mtases in the in vitro study [80]. Fleximers are a class of flexible nucleoside analogs with a split purine nucleobase, which affects the activity of the nucleoside scaffold [81]. A number of fleximers were developed and appeared to be optimal antiviral agents targeting Mtases to block the nucleotide incorporations during the replication of YFV, DENV or ZIKV with low emergence of viral resistances in cell cultures [82,83].

### 4.3. Nucleoside Synthesis Inhibitors

Ribavirin is a broad-spectrum antiviral drug used to treat the infections of various RNA viruses. The inhibitory mechanism for ribavirin involves the inhibition of flaviviral replication through the suppression of guanine nucleotide biosynthesis [84]. Moreover, ribavirin is also believed to be an inhibitor of the viral NS5 RdRp domain involved in the interference of the mRNA capping [85]. Antiviral therapy consisting of ribavirin was shown to be effective on various mosquito-borne flaviviruses such as YFV and DENV in both cell-based systems and animal models. Ribavirin protected YFV-challenged hamsters from lethal infection even when it was administrated two days after virus inoculation [62]. However, the therapeutic effect was quite limited in primates. Modified ribavirin derivatives of ETAR and IM18 manifested better inhibitory effects on DENV2 virus infection by halting the replication potently in cell cultures [86].

Nucleosides of 6-azauridine and its derivatives could perturb pyrimidine biosynthesis, thus suppressing the replication of multiple mosquito-borne flaviviruses including WNV, YFV, DENV, ZIKV and JEV in vitro at micromolar concentrations [87,88,89]. Similar inhibitor of 5-aza-7-deazaguanosine (ZX-2401) was also found to have a synergistic effect when combined with the interferon in the treatment of YFV infection [90].

Inhibitors targeting flaviviral NS5, especially the nucleosides inhibitors targeting viral RdRps are the best studied and understood antiflaviviral agents for their initial application in HCV treatment. The reemployment of these compounds could reduce excessive development costs. Nevertheless, almost all the drugs did not reach the clinical evaluation stage, and the mechanisms of some newly developed inhibitors still need further research for clarification.

## 5. Inhibitors Targeting Host Cell Lipid Metabolism

A series of studies have confirmed the critical roles of cellular lipid metabolism during flaviviral replication and assembly [91]. Flaviviruses utilize the host lipid remodeling to provide energy for their own genome replication and to change membrane fluidity for viral assembly [92,93]. The lipid droplets were also key elements during the nucleocapsid encapsulation [94]. Therefore, the host lipid metabolism could be an optimal target, and a variety of FDA-approved compounds targeting lipid-associated factors are under investigation and evaluation to be alternatives in the development of antiflaviviral agents (Figure 1).

Lipid synthesis plays crucial roles in flaviviral life cycles. Therefore, targeting lipid metabolism modulators has become an optimal strategy to combat viral infection. Sterol regulatory element-binding protein (SREBP) is a critical host factor that regulates the lipid metabolism. A number of SREBP inhibitors such as NDGA (nordihydroguaiaretic acid) and its derivatives, as well as PF-429242 and fatostatin, inhibited viral infection of WNV, DENV and ZIKV potently in cell cultures by interfering the SREBP-dependent lipidomic reprogramming [95]. AMPK (adenosine monophosphate-activated protein kinase) is a main regulator of the host glycolysis and lipid metabolism. Its activators, PF-06409577, metformin and AICAR (5-aminoimidazole-4-carboxamide ribonucleotide) were reported to have antiviral potentials in WNV, DENV and ZIKV infections, abolishing viral replication through the impairment of AMPK phosphorylation [96,97]. In addition, metformin was also found to increase the survival rate of DENV-infected AG129 mice and attenuate severe DENV infection in a retrospective cohort study [98,99]. Acetyl-coenzyme A carboxylase (ACC) is a key enzyme to support the synthesis of fatty acids. Several small-molecule compounds targeting this enzyme were identified to be specific inhibitors against WNV, DENV and ZIKV. One of them, known as PF-05175157, showed a robust protective effect on WNV infection in a mouse model by reducing viremia and viral titers in the targeted organs [100]. Orlistat (tetrahydrolipstatin) was an FDA-approved drug designed to inhibit the enzyme activity of the thioesterase domain from the fatty acid synthase, thus interfering in the synthesis of long-chain fatty acids. This drug was found to have promising activities against eight DENV isolates, ZIKV and JEV, reducing viral infections and productions as well as the genome copy numbers in cell-based systems [101].

Sphingolipids are a class of lipids that consist of long-chain amino alcohol sphingosines. They are abundant especially in the neurocytes, which are also the targets of most mosquito-transmitted flaviviruses. Recent study suggested that a specific inhibitor called GW4869, which blocked the catalyzed conversion from sphingomyelin to ceramide, could reduce viral infections of WNV and ZIKV in human fetal astrocytes [102,103].

Cholesterol is closely involved in the key processes of flavivirus infection including the viral entry, the related innate immunity and the virion production. Statins are a class of compounds that inhibit cholesterol biosynthesis and were found to have antiviral potentials against DENV and ZIKV by competitive inhibition to impede the formation of viral replication complexes, thus reducing the release of infectious virions [104]. Moreover, this class of compounds possesses a pleiotropic antiviral effect observed in various viruses, and the combination of statins with other specific antivirals tend to produce synergistic effect, such as the remarkable activity observed in Ebola patients [105]. Therefore, the supplement of this widely used and safe drug might contribute to antiflaviviral therapy in the future. One of the statins, lovastatin was able to promote the survival of DENV-infected AG129 mice [106]. Due to its excellent safety record and potential of inhibiting inflammation, lovastatin was applied in clinical studies to evaluate its effect on DENV patients. However, the treatment did not realize its efficacy, with no significant decrease of viral RNA levels or symptomatic relief, but only a slight tendency to clear the virus [107]. The cholesterol trafficking inhibitor imipramine and the cholesterol membrane transporter blockers benzamil and ezetimibe were also potent DENV or ZIKV inactivators in a cell-based system [108].

Mosquito-transmitted flaviviruses especially DENV and ZIKV exploit the cellular lipid metabolism to promote their replications, making FDA-approved lipid-lowering drugs to be potential potent host-directed treatment options. However, in vivo and clinical investigations were still limited to verify their prospects for the clinical application.

## 6. Other Antiflaviviral Agents

The efforts for antiflaviviral agent exploration were also made by targeting the viral structural proteins. Heterocyclic compounds such as NITD-448, D02, D04 and D05 were identified to inhibit DENV infection by interfering with E protein-mediated viral cell entry or membrane fusion [40,109]. A natural compound, gossypol, was screened out from a natural product library and exhibited strong inhibitory effect on ZIKV infection of almost all 10 strains by targeting the envelope protein domain III [110]. This compound was also confirmed to suppress DENV of all four serotypes in cell cultures [110]. A ZIKV envelope-derived peptide Z2 was demonstrated to inhibit ZIKV and other flaviviruses such as YFV and DENV by binding to viral E proteins to inactivate the virions [111]. Moreover, it could also block the vertical transmission in ZIKV-infected pregnant mice and protected mice from fatal virus challenges [111]. ST-148 was a compound designed to block the viral capsids. It was reported that the compound reduced the viral cytopathic effects and suppressed DENV infection of all four serotypes [112].

Arbidol (umifenovir) is a licensed antiviral drug used to treat influenza in China and Russia [113]. It has a broad-spectrum inhibitory effect on various DNA and RNA viruses including some of the mosquito-transmitted viruses. Arbidol impeded WNV and ZIKV infections at micromolar concentrations in Vero cells specifically by interfering with virion–cell interactions [114]. Another clinically used agent, methotrexate (MTX), was also found to possess anti-ZIKV activity in vitro by the inhibition of dihydrofolate reductase (DHFR), a key enzyme for the de novo synthesis of purines and pyrimidines [115]. Enoxacin is an FDA-approved fluoroquinolone with significant anti-DENV and ZIKV potencies at low micromolar concentrations, and it also protected ZIKV-infected mice by lowering the viral loads in the serum and targeted organs in the treatment group [116].

The small-molecule inhibitor NGI-1 is a modulator of oligosaccharyltransferase (OST), which is a critical host factor during flaviviral infection, playing an important role in the formation of the viral replication complex. NGI-1 was reported to have pan-flavivirus antiviral activity and block viral RNA replication of DENV and ZIKV in multiple disease-relevant cell lines [117]. Trametinib, a mitogen-activated protein kinase kinase (MEK) inhibitor was identified to block the replications of YFV, DENV and ZIKV with high efficiency in cell-based systems [118]. Estrogen receptor modulators such as raloxifene hydrochloride and quinestrol were demonstrated to be effective inhibitors of WNV, DENV and ZIKV infections at micromolar concentrations with low cytotoxicity in various cell types [119]. Alkaloid anisomycin is a natural compound originally identified as an antifungal drug. Researchers found that it inhibited the infection of all DENV serotypes and ZIKV of Asian and African strains during viral replication at noncytotoxic concentrations in both Vero cells and human cell lines [120]. A low dose of anisomycin might also provide protection against ZIKV challenge in a mouse model [116]. Synthesized retinoid N-(4-hydroxyphenyl)-retinamide (4-HPR or fenretinide) was an antiflaviviral compound that covered WNV, DENV and ZIKV [121]. It reduced the steady state of the viral genomic RNA accumulation, and the viremia in mice challenged by DENV was decreased when administered with the drug orally [122].

## 7. Conclusions

Mosquito-transmitted flavivirus infection is still a major public issue and heavy burden worldwide. Vaccines are only available for some of these viruses, and the broad application in endemic areas is quite unsatisfying. Given the tremendous impact of flaviviral infection on public health and rare progress in vaccine development, there is an urgent need for direct-acting antivirals with high efficiency for both prophylactic and therapeutic activity against the infections of mosquito-borne flaviviruses. Despite numerous efforts having been made to develop novel and optimal antiviral drugs against these flaviviruses, there is currently no pharmacological therapy approved to specifically treat the infections of these viruses, and small molecular inhibitors have rarely progressed into early clinical investigations (Table 1). Research needs to advance to in vivo studies and clinical trials quickly if appropriate. Reemployment and repurposing approaches could be promising strategies to develop optimal antiflaviviral agents, and the lessons learned from those failing therapeutic trials will contribute to future studies during clinical assessments of potential novel compounds against flaviviral infection. In addition, the significant advances in molecular and structural virology as well as the scientific methods on inhibitors screening and studying, which target the critical host or viral elements involved in the flaviviral life cycle, provide highlights and prospects for the advancement of the future medical therapy for mosquito-borne flaviviruses.

## Figures and Tables

**Table 1:** The process of viral targets for antiflaviviral agents with their development stage.

Target	Classification	Representatives of the Compounds	Flaviviruses	Study Stages	References
NS2B–NS3 protease	Peptidomimetic inhibitors	Agmatine-based peptidomimetics	WNV	Cell culture	[14]
		Retro-tripeptides	DENV	Cell culture	[13]
		Fused bicyclic peptides of imidazolidinones and pyrrolidines	DENV2	Cell culture	[15]
		Peptidomimetics consisting of Abz-Arg-Arg-Arg-Arg-X-X-X-X-Tyr(NO_2_)-NH_2_	DENV1, DENV2, DENV3, DENV4	Cell culture	[16]
		Macrocyclic peptidomimetics	DENV2, ZIKV	Cell culture	[17,18]
		Metallopeptidomimetic compounds	WNV, ZIKV	Cell culture	[19]
	Peptide-based hybrids inhibitors	Peptide hybrids containing thiazolidine or thiophene	WNV, DENV2	Cell culture	[20]
		Dipeptides containing boronic acid or its derivatives	WNV, DENV2, ZIKV	Cell culture	[21]
		Tripeptides composed of Bz-X-Lys-Phg-NH_2_ or phenacetyl substituents	WNV, DENV3	Cell culture	[22,23]
		Synthesized tetrapeptide aldehyde compounds	WNV	Cell culture	[24]
		Peptidyl-aldehyde compounds	WNV, DENV2, ZIKV	Cell culture	[25,26]
	Synthetic or natural compounds	Synthesized compounds targeting histone-modifying enzymes	WNV, DENV, ZIKV	Cell culture, animal model	[27]
		Ivermectin, tyrothricin and selamectin alexidine	WNV, DENV	Cell culture	[28]
		Aprotinin	DENV, ZIKV	Cell culture	[29,30]
NTPase and helicase	□	Suramin	DENV	Cell culture	[32]
		Benzothiazole and pyrrolone	WNV, DENV	Cell culture	[33]
		FSBI, 1H-benzotriazole and 1H-benzimidazole analogs	WNV, DENV, JEV	Cell culture	[34,35,36]
NS4	□	NITD-618	DENV	Cell culture	[38]
		CCG-3394 and CCG-4088	DENV	Cell culture	[39,40]
		Lycorine	WNV, YFV, DENV	Cell culture	[41]
NS5-RdRp	Nucleoside inhibitors	R-1479 and RO-9187	WNV	Cell culture	[47]
		GS-441524	YFV, DENV	Cell culture	[48]
		2′-C-Methylated nucleosides	DENV	Cell culture	[49,50]
		INX-08189	DENV2	Cell culture	[51]
		Tritylated pyrimidine nucleosides	YFV, DENV2	Cell culture	[52]
		2′-C-Methylated nucleosides	ZIKV	Cell culture	[53]
		2′-C-Methylcytidine, 7-deaza-2′-C-methyladenosine	WNV, YFV, DENV	Animal model	[47]
		2′-C-Ethynyl-substituted nucleosides and derivatives	WNV, YFV, DENV, ZIKV	Cell culture, animal model	[54,55,56,57]
		BCX4430	WNV, YFV, DENV, ZIKV, JEV	Cell culture	[58,59,60,61]
		T-1106	YFV	Animal model	[62]
		T-705	WNV, YFV, ZIKV	Cell culture, animal model	[63,64]
		Sofosbuvir	YFV, DENV, ZIKV	Animal model	[66,67,68,69]
		Balapiravir	DENV	Phase I	[70]
	Non-nucleoside inhibitors	Pyridobenzothiazole-based compounds	WNV, DENV	Cell culture	[72,73]
		DENV allosteric N-pocket inhibitors	DENV	Cell culture	[74,75]
		Rifapentine	YFV	Animal model	[77]
NS5-Mtase	□	SAH, sinefungin, GMP	DENV, ZIKV	Cell culture	[78]
		GRL-002 and GRL-003	WNV	Cell culture	[79]
		5′-Silylated 3′-azidothymidine substituents	WNV, DENV	Cell culture	[80]
		Fleximers	YFV, DENV, ZIKV	Cell culture	[82,83]
Nucleoside synthesis	□	Ribavirin	YFV, DENV	Animal model	[62]
		Modified ribavirin derivatives of ETAR and IM18	DENV2	Cell culture	[86]
		6-Azauridine and its derivatives	WNV, YFV, DENV, ZIKV, JEV	Cell culture	[87,88,89]
		ZX-2401	YFV	Cell culture	[90]
Host cell lipid metabolism	Lipid synthesis	NDGA and its derivatives, PF-429242, fatostatin	WNV, DENV, ZIKV	Cell culture	[95]
		PF-06409577, metformin, AICAR	WNV, DENV, ZIKV	Cell culture, animal model, clinical evaluation	[96,97,98,99]
		PF-05175157	WNV	Animal model	[100]
		Orlistat	DENV, ZIKV, JEV	Cell culture	[101]
	Sphingolipids	GW4869	WNV, ZIKV	Cell culture	[102,103]
	Cholesterol	Statins	DENV, ZIKV	Cell culture, animal model, clinical evaluation	[104,105,106,107]
		Imipramine, benzamil, ezetimibe	DENV, ZIKV	Cell culture	[108]
Viral structural proteins	Viral E protein	NITD-448 and D02, D04 and D05	DENV	Cell culture	[40,109]
		Gossypol	DENV, ZIKV	Cell culture	[110]
		Z2	YFV, DENV, ZIKV	Cell culture, animal model	[111]
	Viral C protein	ST-148	DENV	Cell culture	[112]
Licensed drugs	Licensed drugs	Arbidol	WNV, ZIKV	Cell culture	[114]
		MTX	ZIKV	Cell culture	[115]
		Enoxacin	DENV, ZIKV	Cell culture, animal model	[116]
Other compounds	Small-molecule inhibitor	NGI-1	DENV, ZIKV	Cell culture	[117]
	MEK inhibitor	Trametinib	YFV, DENV, ZIKV	Cell culture	[118]
	Estrogen receptor modulators	Raloxifene hydrochloride and quinestrol	WNV, DENV, ZIKV	Cell culture	[119]
	Natural compound	Alkaloid anisomycin	DENV, ZIKV	Cell culture, animal model	[120]
	Synthetic compound	Fenretinide	WNV, DENV, ZIKV	Cell culture, animal model	[121,122]
